# The integrated on-chip isolation and detection of circulating tumour cells

**DOI:** 10.1039/d3sd00302g

**Published:** 2024-03-26

**Authors:** Sophia M. Abusamra, Robert Barber, Mohamed Sharafeldin, Claire M. Edwards, Jason J. Davis

**Affiliations:** a Nuffield Department of Surgical Sciences, University of Oxford Oxford OX3 9DU UK; b Department of Chemistry, University of Oxford Oxford OX1 3QZ UK jason.davis@chem.ox.ac.uk; c Department of Chemistry, University of Otago Dunedin 9016 New Zealand; d Nuffield Department of Orthopaedics, Rheumatology and Musculoskeletal Systems, University of Oxford Oxford UK

## Abstract

Circulating tumour cells (CTCs) are cancer cells shed from a primary tumour which intravasate into the blood stream and have the potential to extravasate into distant tissues, seeding metastatic lesions. As such, they can offer important insight into cancer progression with their presence generally associated with a poor prognosis. The detection and enumeration of CTCs is, therefore, critical to guiding clinical decisions during treatment and providing information on disease state. CTC isolation has been investigated using a plethora of methodologies, of which immunomagnetic capture and microfluidic size-based filtration are the most impactful to date. However, the isolation and detection of CTCs from whole blood comes with many technical barriers, such as those presented by the phenotypic heterogeneity of cell surface markers, with morphological similarity to healthy blood cells, and their low relative abundance (∼1 CTC/1 billion blood cells). At present, the majority of reported methods dissociate CTC isolation from detection, a workflow which undoubtedly contributes to loss from an already sparse population. This review focuses on developments wherein isolation and detection have been integrated into a single-step, microfluidic configuration, reducing CTC loss, increasing throughput, and enabling an on-chip CTC analysis with minimal operator intervention. Particular attention is given to immune-affinity, microfluidic CTC isolation, coupled to optical, physical, and electrochemical CTC detection (quantitative or otherwise).

## Abstract 2

Accessible microfluidic configurations support CTC isolation (by size, rigidity, antigen expression) and detection in a single platform. Integrated detection can be achieved through optical, electrochemical, and physical means.

## Graphical abstract

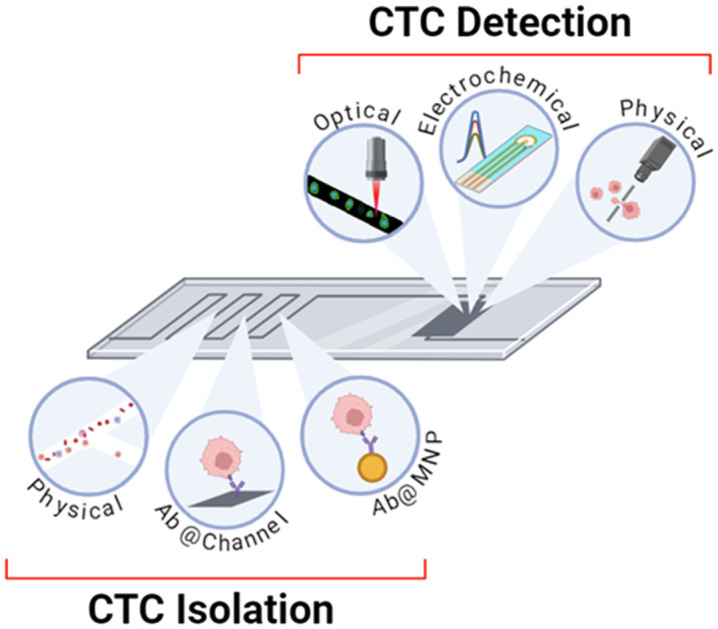

## 1 Introduction

Cancer cells that detach from a primary tumour or metastatic site and circulate though the bloodstream are known as circulating tumour cells (CTCs), and represent a promising target for non-invasive tumour sampling. Despite an established clinical and biological relevance, a cost effective, robust, and rapid method of assaying these with sufficient sensitivity has yet to materialise. CTCs are highly associated with disease metastasis, yet the first FDA-approved method, CellSearch®, has been shown to detect them in only 57% of patients with metastatic disease, with a prognostic cut-off of >5 CTCs/7.5 mL of blood.^1^ Therefore, substantial and impactful improvements to address both practical applicability (cost, blood volumes *etc.*, see below), and the high proportion of patients with metastatic disease who are likely below this threshold, are needed.

CTC analysis is comprised of two crucial steps: isolation (specific capture from within an enormous excess of blood cells), and subsequent detection (quantification and/or identification of captured cells). Nearly all reported platforms, including CellSearch®, separate these two processes, despite it being known that sample transfer between dissociated isolation and detection contributes to cell loss and increased cost, time, and labour.^2–5^ As such, there is significant interest in integrated platforms that enable a rapid, sensitive, and streamlined analysis from low blood volumes. This review will focus on the state of the art on-chip technologies that integrate CTC isolation and detection into a single, and potentially highly scalable, microfluidic device.

### 1.1 Biological significance and clinical utility of CTCs

**Fig. 1:**
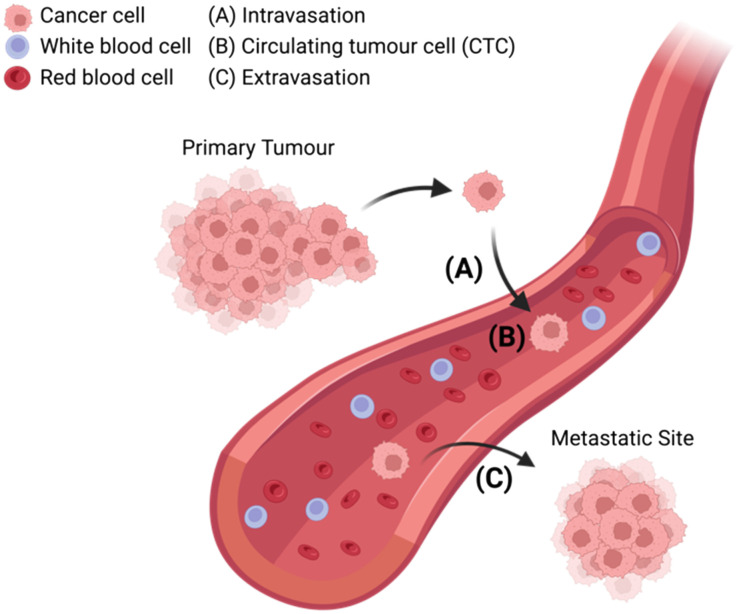
Schematic representation of circulating tumour cells (CTCs) breaking off from the primary tumour site and (A) intravasating into circulation, (B) traveling through the bloodstream among red blood cells and white blood cells, (C) extravasating from circulation with the potential to seed sites of cancer metastasis in distant tissue.

The metastasis of cancer from primary tumour to distal tissues is associated with approximately 90% of cancer deaths, of which there were almost 10 million in 2020.^6^ The lethality of metastasis is grimly indicated by the drastic difference in five-year survival rates between patients with localised and distant disease (99% *versus* 27%).^7^ Thus, understanding how cancer cells transform between primary and metastatic sites is crucial if one seeks to prevent disease progression and guide treatment decisions.^8^ At present, it is known that CTCs are shed from the primary tumour and intravasate into circulation, where they can potentially extravasate into distant tissues and seed secondary sites of disease (Fig. 1). However, further understanding is required to elucidate disease dissemination and metastasis mechanisms; understanding which could be provided through improved CTC analyses.^9–12^

The isolation of CTCs from whole blood is a form of liquid biopsy, and as such offers great clinical utility, as it is less invasive (accessible *via* peripheral venous phlebotomy) and less expensive than solid tissue biopsies (performed by highly trained professionals and may require radiographic guidance).^13,14^ Furthermore, solid tissue biopsies can easily miss small cancerous lesions (especially in early-stage disease), and may fail to capture intra-tumour heterogeneity and intra-patient tumour evolution with time.^15–17^ CTCs have also been shown to change in a more dynamic way than other serum cancer biomarkers such as PSA, AFP, CA-199, and CEA.^18,19^ They provide, then, a more accurate and up to date clinical picture of disease, in capturing a ‘snapshot’ of the tumour condition,^9,20^ representing a validated prognostic marker for numerous malignancies.^21–26^ While other methods of liquid biopsy including circulating tumour DNA/cell-free DNA (ctDNA/cfDNA) and exosomes have been described, CTCs offer innate advantages with the opportunity to study whole cells, as they can be cultured for *in vivo* and functional studies.^27–29^ Furthermore, CTCs allow for protein- and RNA-based molecular profiling and are certain to be of tumour origin, unlike ctDNA/cfDNA, wherein DNA analyses can be confounded by clonal haematopoietic mutations of indeterminate potential (CHIP).^27–32^

The biological significance of CTCs is most clearly demonstrated by the very direct association of increased CTC load (higher haematological abundance) with poor prognosis and reduced overall disease-free survival.^33–38^ In addition to enumeration, these cells hold a wealth of biological information that can be unlocked *via* genetic profiling. Recent studies have focused on genomic sequencing of CTCs to reveal their neoplastic origin, elucidating clonal evolution and offering insight into metastatic pathways.^39–41^ This downstream genetic profiling also has the potential to predict response to therapy and to risk stratify patients, guiding treatment decisions.^42–50^

### 1.2 Microfluidics (μFs) for CTC isolation & detection

**Fig. 2:**
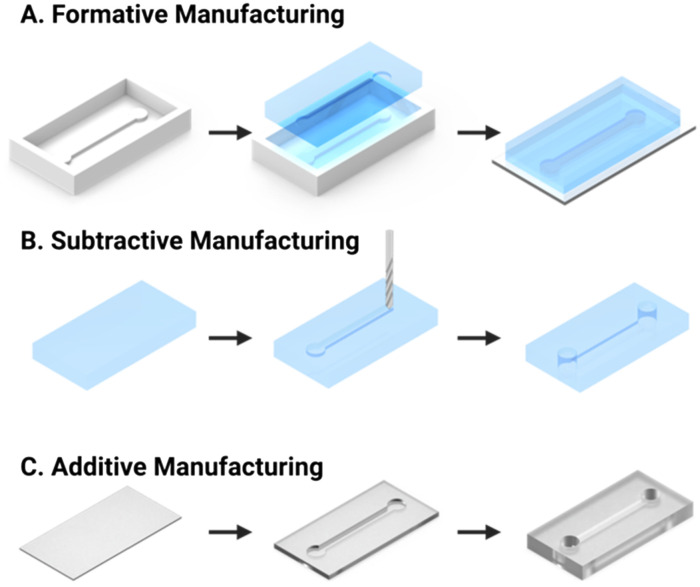
Schematic representation of the most common microfluidic fabrication strategies. (A) Formative manufacture is achieved by casting of optically transparent PDMS into a mould. The chip is then formed by mounting the resultant PDMS cast piece onto a glass slide. (B) Subtractive manufacture is performed *via* computer numerical control (CNC) milling of a solid substrate. (C) Additive manufacture utilises layer by layer selective curing of transparent resin to assemble the chip and internal channel (*e.g.*, SLA or DLP 3D printing).

Microfluidics (μFs) is a multidisciplinary field that enables the complex manipulation of microlitre volumes of reagents and as such, has demonstrated applicability to numerous areas of research, including drug discovery, cell culture, and personalised medicine.^51,52^ The microchannel configurations of microfluidic chips can be designed with diverse geometries to achieve a range of tasks including reagent delivery, mixing, or extraction of specific targets.^36^ Historically, formative manufacturing has been the default microfluidic fabrication technique, wherein a liquid polymer precursor (*e.g.*, polydimethylsiloxane, PDMS) is cast and cured into a photolithographically fabricated mould (Fig. 2A). Conversely, subtractive manufacturing involves the selective removal of substrate material *via* laser ablation or computer numerical control (CNC) milling to form a channel (Fig. 2B). Recently, the decreasing cost of high-resolution desktop 3D printers using stereolithography (SLA) through point-by-point laser scanning or digital light processing (DLP) techniques have enabled in-house additive manufacturing of microfluidic channels with features down to 25 μm (Fig. 2C). These methods use a laser or UV light source to selectively polymerise a liquid resin into the desired solid geometry at very low cost (around £0.03–0.15 per cm^3^). 3D printing with inherently non-cytotoxic materials or the post-cure application of a protective coating (*e.g.*, bovine serum albumin, BSA) has allowed for the fabrication of biocompatible microfluidic chips.^53^

Microfluidic platforms, or “chips” have been extensively applied to the challenge of CTC isolation, using both immunoaffinity and physical methods, as discussed in sections 2.1 and 2.2, respectively. They present an opportunity for a simplified and miniaturised workflow, requiring less time (<5 hours) and sample volume (sub mL)^54–56^ than the CellSearch® commercialised standard for CTC enrichment (7.5 mL).^57–60^ The detection of CTCs in microfluidic devices has most frequently been achieved using optical microscopy methods (section 3.2), mandating good optical transparency of the platform. For this reason, transparent PDMS substrates, and photo-curing resins, are ubiquitous.

### 1.3 On-chip integration of CTC isolation and detection

As mentioned above, CTCs analyses include two critical steps: isolation and detection. Isolation is the separation of tumour cells from blood, with detection being either a subsequent quantification (*i.e.*, electrochemical or optical enumeration) or identification (*i.e.*, confirmation that captured cells are CTCs and not an erroneously captured cell).

**Fig. 3:**
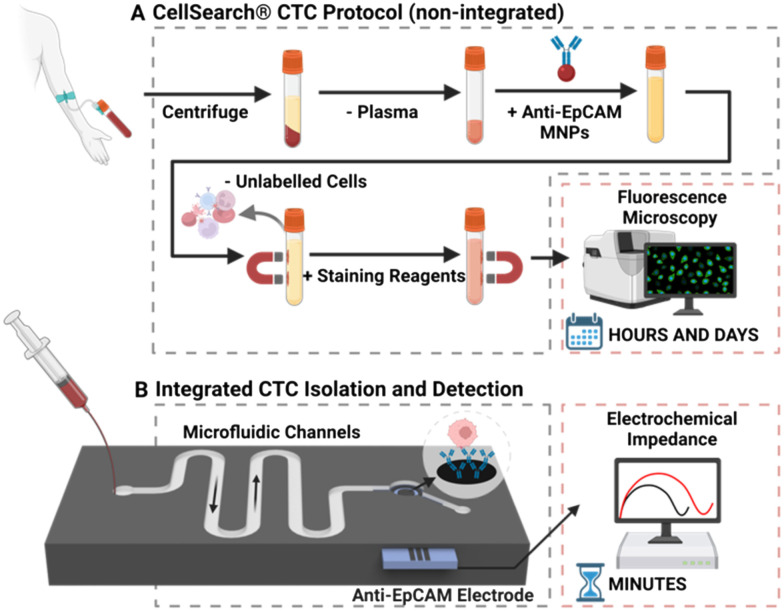
(A) Schematic representation of the CellSearch® platform. Firstly, some 7.5 ml of whole blood is centrifuged to remove plasma, the residual volume is then mixed with anti-EpCAM modified magnetic nanoparticles (MNPs) to separate CTCs from healthy cells. The isolated cells are then enumerated using immunofluorescence microscopy. (B) Representative schematic of a configuration that facilitates CTC isolation and electrochemical (*e.g.*, impedance) detection within a single microfluidic chip.

The majority of reported methods of CTC isolation, including CellSearch®, necessitate the elution and manual transfer of isolated cells to a separate module or device for detection (schematised in Fig. 3A).^61–65^ CTCs can be lost during this transfer *via* adhesion to pipette tips and sample tubes across experimental steps, or simple retention within the device or chamber.^2,3^ Such manual transfer increases time and labour, introduces inter-test variability, adversely affects assay accuracy, and can inflict cell damage.^4,5^ The latter arises because human cells are mechanically fragile, deformable, and sensitive to environmental change (and thus transport/manipulation can affect sample quality).^66,67^ As CTCs need to be in optimal condition for potential downstream analysis, such as genomic interrogation or culture, each damaged cell represents the loss or tarnishing of potentially valuable information. In response to such issues, a low but growing number of reports have integrated CTC isolation and detection into a single microfluidic chip, as summarised in Table 1 and described as “integrated” methods from hereon. These methods require less time to operate, reducing assay cost, curtailing opportunities for cell loss, and necessitating notably lower sample volumes.^52,68,69^ They facilitate convenient sample processing wherein multiple experimental steps can be completed on-chip without manual transfer.^70^ Such integrated approaches have been shown to support a robust sensitivity (detection limit of <10 cells per mL) in a scalable, fast (<1 hour per sample) system capable of analysing CTCs in blood volumes as low as 1 mL.^71–73^

**Table 1:** Summary of fully integrated methodologies of CTC isolation and detection discussed within this review, as displayed according to the differing methods of isolation and detection utilised. Categories of isolation utilised in integrated platforms include antigen-independent (physical) and antigen-dependent (immunoaffinity) based isolation. Methods of detection employed in integrated platforms discussed can be categorised as electrochemical, optical, and/or physical

	Integrated detection method												
			Electrochemical detection			Optical detection							Physical and optical detection
			Amperometry	Impedance	Voltammetric pulse	UCL imaging	RIDA	*In situ* Raman barcoding	Brightfield microscopy	Immuno-blotting	IF	Deformability cytometry	Cavitation inception pressure
Integrated isolation method	Antigen-independent (physical) isolation	Size-based exclusion with mesh or microfilter								41^b^	137		
		Size-based sorting in trap channel or hole array						113^b^	142^b^		55	138	
											139		
		DEP	54	146^b^					142^b^				
		Hydrodynamic separation with chip geometry		146^b^			120^b^			41^b^			
		Hydrodynamic cavitation											14
	Antigen-dependent isolation	Immobilized Ab on-chip		112^a^	114						116		
				119^a^	112^a^						119^a^		
											56		
		Off-chip incubation with Ab-conjugated NPs			117	118	120^b^				121		
											115		
											122		
		Ab-conjugated Raman active nanoprobes						113^b^					

a Platforms that use multiple detection methods.

b Platforms that use multiple isolation methods.

Within integrated platforms, CTCs can either be isolated and detected on the same surface (*e.g.*, isolation of CTCs using a filter mesh followed by immunofluorescence staining on the same mesh), or flow within the same chip from isolation to detection regions (*e.g.*, CTC isolation on channel walls with immobilised antibodies, followed by direct flow of captured cells to an electrochemical detection module), as schematised in Fig. 3B. Within this review, we provide an overview of common approaches for CTC isolation and elaborate on the implementation of such methods within integrated systems. Subsequently, strategies for CTC detection are summarised and the coupling of such methods on integrated platforms is detailed.

## 2 CTC isolation

As discussed, integrated platforms for CTC analysis (summarised in Table 1) both isolate and detect these rare cells on a single microfluidic chip. Isolation from whole blood is challenging, with the most significant barriers being the low abundance,^74,75^ morphological similarity to white blood cells (WBCs),^76,77^ and phenotypic heterogeneity.^78^ Nonetheless, CTC isolation has been demonstrated using many proof of principle methods, broadly divisible into two approaches: antigen-dependent (immunoaffinity; *e.g.*, antibody-loaded magnetic nanoparticles or other solid supports), and antigen-independent (*i.e.*, separation through physical properties).^37,76,79^ Section 2 will overview current immunoaffinity and physical approaches to CTC isolation (see section 2.1 and section 2.2, respectively) and detail how these approaches are applied within integrated platforms (see section 2.1.1 and section 2.2.1).

To evaluate the performance of CTC isolation techniques, the following parameters are considered: purity, enrichment or capture efficiency, and throughput (or turnaround time/TAT).^33^ Purity describes the specific capture of CTCs from a heterogeneous background of other cells, while enrichment refers to the ability to increase the proportion of CTCs within a given sample. Capture efficiency is a metric presented to summarise the number of CTCs captured out of a known population (as a percentage), and the amount (in volume or number of samples) that can be processed per a given amount of time is known as throughput/TAT.^33^

### 2.1 Antigen-dependant isolation

Antigen-dependent (immunoaffinity-based) methods of isolation utilise antigen recognition to select for or against cells based on markers present on the cell membrane, achieved by immobilising a complimentary antibody or aptamer on a supporting structure.^80,81^ Positive enrichment selects for CTCs; conversely, negative enrichment excludes non-CTCs.^12^ These strategies often target epithelial cell adhesion molecule (EpCAM/CD326), as a high proportion (up to 90%) of cancers are of epithelial origin and thus express this marker.^12,82–84^

As an exemplar, the CellSearch® platform mentioned in section 1 uses anti-EpCAM conjugated magnetic nanoparticles (MNPs) to positively select for EpCAM^positive^ cells (Fig. 3A). The protocol results in a low purity output (60–70%) due to a high residual leucocyte population of ∼1000–3000 cells from the 7.5 mL sample.^85–87^ This background noise necessitates the use of immunofluorescent (IF) imaging of cytoplasmic and cell surface markers to detect and enumerate CTCs, resulting in a high instrument cost (∼£200 000), with cost per test ∼£800 and TAT ∼5 hours.^88–90^

Relying on positive enrichment alone may erroneously include non-tumour EpCAM^positive^ cells and fail to address the dynamic expression of cell surface antigens.^39,91–93^ CTCs are known to undergo an epithelial-to-mesenchymal transition (EMT) while in circulation, during which they acquire the migratory properties typical of mesenchymal cells, lose their epithelial characteristics and downregulate EpCAM expression.^79,94^ Attempts to mitigate this downregulation have sought to diversify the surface antigens used in antigen-dependant CTC capture by including mesenchymal (*e.g.*, vimentin^positive^) and pseudo-endothelial (*e.g.*, dual EpCAM^positive^ and CD31^positive^) markers.^39,68,95,96^ Targeting of malignancy specific markers has also been investigated to address this challenge, including positive selection for prostate specific membrane antigen (PSMA) in prostate cancer and for human epidermal growth factor 2 (HER2) in breast and gastric cancers.^22,97^

The inverse immune-affinity approach, negative enrichment, makes use of antibody-coated solid supports to target cell surface antigens such as CD45 (specific to white blood cells) to deplete healthy blood cells from a sample.^98,99^ A key advantage of negative enrichment is that intact (label-free) CTCs are obtained independent of their specific antigen expression, and thus, a heterogenous population of CTCs can be isolated (*i.e.*, including low-EpCAM-expressing CTCs).^100,101^ This approach does not, however, afford a tumour-specific selection and thus typically results in a low purity (as exemplified by the low and inconsistent purities across different systems varying between 0.97–10%, going up to a maximum of 34.8 ± 14%).^102–111^

#### 2.1.1 Applying antigen-dependent isolation in fully integrated platforms

Antigen-dependant methods of CTC isolation have been successfully deployed in integrated platforms (section 1.3), exemplars of which are herein discussed. Reflecting the trend of capturing CTCs using markers other than EpCAM to overcome phenotypic heterogeneity, integrated platforms have targeted cluster of differentiation 36 (CD36, a metastatic marker),^112^ cluster of differentiation 133 (CD133),^113^ melanocortin 1 receptor (MC1R),^114^ N-cadherin,^115^ and mucin1 (MUC1).^113^ These affinity based isolations are achieved by immobilising antibodies on the inner surfaces of microfluidic channels^54,112,116^ or tethering antibodies to magnetic nanoparticles (MNPs) which can be magnetically manipulated within the chip.^117,118^ Such approaches have enabled CTC isolation from relatively small volumes of patient blood samples (1 mL for MNP-based methods,^115^ 2 mL for immobilised-Ab methods^119^). MNPs offer a much larger active surface area than channel walls for the tethering of the desired antibody. In practice, filling a circular 40 μL channel with 10 mg mL^−1^ of MNPs of 200 nm diameter increases the available surface area for antibody immobilisation from 1.6 cm^2^ on the channel walls to 23.2 cm^2^ on the MNP surfaces (>1 order of magnitude greater surface area), potentially greatly improving capture efficacy.

Integrated platforms using Ab-functionalised microfluidic channels capture CTCs on the channel walls by flowing a cell-spiked matrix through the device, while endogenous or exogenous healthy cells continued unimpeded to the channel outlet.^56,112,114,116,119^ These reports have demonstrated capture efficiencies of 72–88% from whole blood, and purities of 85–99.6%.^56,116,119^ Both IF staining^56,116^ and electrochemical detection methods (based on impedance or voltammetry^112,114,119^) have been effectively integrated with upstream channel-wall-immobilised antibody methods of isolation, either by directly imaging the captured CTCs within the channels or probing perturbations to electrode impedance induced by CTC/antibody binding.

One such integrated platform described a dislocated herringbone microfluidic channel modified with anti-EpCAM antibodies.^116^ This channel geometry caused increased turbulence in the flowing sample which increased the likelihood for CTC immunocapture. The authors reported a capture efficiency of 87% using a lung cancer CTC model (H1975) in whole blood, without the addition of any nanoparticle species. The H1975 cells were subsequently enumerated by conducting IF staining and imaging for CK18^positive^ and CD45 (leukocyte marker)^negative^ directly on the chip (with captured and stained cells inside).

**Fig. 4:**
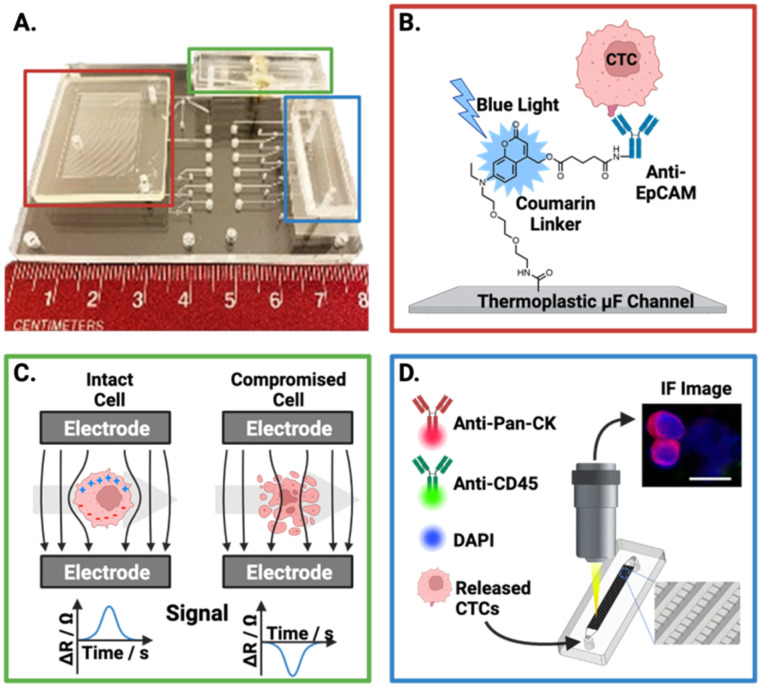
Representative example of an affinity-based CTC isolation and detection system, wherein the principles behind the three SMART-chip modules are schematised. (A) Image of the SMART-chip with each module highlighted. (B) CTC selection module; CTCs are enriched from a flowing blood sample by anti-EpCAM antibodies tethered to a microfluidic channel, the channel is then flushed with buffer to remove unwanted cell background. Isolated CTCs are then released by cleaving the coumarin-based linker with blue light. (C) Impedance module; isolated CTCs proceed to an impedance-based cell enumeration channel to confirm viability. This module had Pt electrodes situation orthogonal to the fluidic channel with ∼50 μm distance. Cells with intact membranes have a higher resistance than the buffer (Δ*R*) and yield positive polarity signals, with dead cells apparently yielding negative signals. (D) Imaging module; the enriched cell population is stained and imaged using a selection of fluorescent antibodies. Reproduced with permission.^119^

Importantly, a controlled release of CTCs following antibody-based capture is critical for downstream analysis. However, the release of viable cells is a challenge for antigen-dependent isolation methods. Pahattuge *et al.* demonstrated the successful CTC release from an isolation channel on a SMART-chip (Fig. 4B).^119^ This was achieved by exposure of the transparent channel to blue light (400–450 nm), photocleaving a coumarin-based linker which was used to immobilise antibodies onto the channel walls, achieving a 90% release. The controlled elution enabled captured CTCs to proceed for viability analysis (Fig. 4C) and immunofluorescent staining (Fig. 4D) within the respective downstream modules, a significant achievement in an 80 mm platform.

One unique report of an affinity-based CTC isolation in an integrated device uses neither channel-immobilised Abs nor Ab-conjugated MNPs, but rather Ab-conjugated Raman active nanoprobes to capture CTCs.^113^ In this work, Cho *et al.* triple functionalised a suite of gold nanoparticles (Au-NPs) for CTC isolation and detection. The bare Au-NPs were modified with biotinylated dsDNA (enabling their capture on the streptavidin pillars), unique Raman reporters (later used for CTC detection, as discussed in section 3.2.1) and a corresponding CTC specific antibody (CD133, HER2, EGFR, EpCAM, or MUC1), forming 5 subsets of labelled Au-NPs. The group described a microfluidic chip with 5 μm-wide micro-pillars to complete size-based exclusion of RBCs and WBCs. A turbulent state of sample flow within the chip facilitated cell–particle mixing. The streptavidin-rich pillar surfaces passively recruited the biotinylated, Raman barcoded CTCs, capturing them within the chip. While this method was not applied to cancer patient blood samples, it was reported to achieve 90% capture efficiency of model CTCs spiked in 4 mL of healthy human blood. However, this required significant pre-treatment of the samples to first isolate mononuclear cells using a density gradient Ficoll-Paque protocol involving dilution, centrifugation, washing and separation.

While the chemical (biotin–streptavidin) mediated capture of CTCs within the chip in the above report was reversible by enzymatic cleaving of a dsDNA linker, Au-NPs cannot be magnetically manipulated. An alternative, would be, of course, to employ antibody-MNPs. CTC–MNP complexes can be simply isolated and released from the running solution by application or removal of a permanent or modulating electromagnetic field. This enables the controlled retention of CTCs within the channel, while other contaminants are eluted from the device and eventually released into a clean running buffer, as required.^117,118^

These Ab-modified MNP approaches have reported capture efficiencies of 65–99.9% from whole blood. To do so, microfluidic platforms have utilised various channel designs to achieve passive mixing and thereby, maximum capture.^115,117,118,120–122^ These include serpentine and herringbone geometries, both of which have been shown to disturb laminar flow within the channel to improve mixing efficiency beyond what would be possible by diffusion alone.^123^ One study noted ∼80 WBCs captured along with CTCs from 1 mL of whole blood, a considerable reduction from ∼10 000 WBCs per 7.5 mL of blood recorded for the isolation method used by CellSearch®.^117^

While most reports using Ab-conjugated MNPs utilise a constant magnetic field and a single molecular target,^117,118,121^ recent fully integrated platforms have included progressively more sophisticated techniques, selecting for multiple cell surface markers and ranking captured cells based on magnetic gradients. An example which exploits the power of full integration is one that achieved CTC isolation and ranked these based on surface antigen expression. Poudineh and colleagues, demonstrated their magnetic ranking cytometry (MagRC) microfluidic chip, featuring a magnetic field gradient along a channel to sort cells based on magnetisation. The amount of nanoparticle loading on cells was, specifically, reflective of the degree of expression of the target antigen, which allowed for instantaneous on-chip CTC profiling. The group reported a capture efficiency of 90–97% and an integrated IF staining based detection limit of 1–10 cells per mL.^115^

**Fig. 5:**
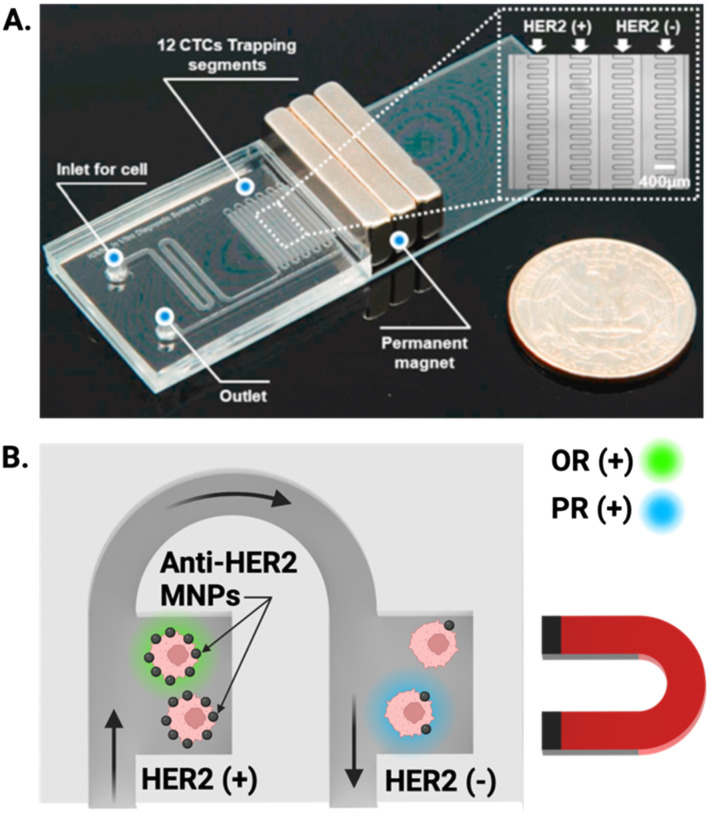
Representative example of MNP assisted isolation of CTCs in an integrated microfluidic chip. (A) Image of a PDMS microfluidic cell sorter which enabled the specific labelling of HER2 receptors with anti-HER2 coated MNPs under the influence of an external magnetic field. (B) Schematic showing magnetically trapped CTCs IF visualised on chip by flowing reagents through the channel for the oestrogen (OR) and progesterone (PR) receptors. Reproduced with permission.^122^

A similar demonstration of this graduation strategy by Lee *et al.* employed MNPs conjugated to anti-HER2 antibodies to target breast cancer cells; once captured from culture media (capture efficiency >99%), CTCs were sorted based on a magnetic gradient into ‘HER2 positive’ and ‘HER2 negative’ regions (Fig. 5A), allowing near instantaneous grading and readout of CTC phenotype based on the expression of the oestrogen receptor (OR) and progesterone receptor (PR). This is particularly impactful as these biomarkers can aid in the prediction of tumour metastasis (Fig. 5B).^122^ Both of these integrated ranking reports utilised the variance in magnetic properties in MNP–CTC complexes to gain insight into phenotypic profiles of the captured CTC population and fully integrated isolation and IF staining. To date, there is only one report which employed an electrochemical method of CTC detection after upstream Ab-MNP assisted isolation.^117^ It can be conjectured that this is because IF can be conducted on captured CTCs without removal of MNPs, whereas introducing MNPs to one of the alternative detection methods discussed in section 3.1 potentially requires CTC separation from MNPs prior to quantification.

While the release of CTCs from Ab-functionalised MNPs presents a challenge, it has been documented, for example, by Wang and colleagues who released 80% of captured CTCs from their anti-EpCAM modified MNPs and showed successful proliferation for 7 days in culture.^118^ Similarly, Poudineh *et al.* achieved a 92% release efficiency, of which 98% of the cells were viable.^115^ Meanwhile, the above discussed report by Pahattuge *et al.* achieved a release of 90% with their photocleavable linker.

In summary, antigen-dependent methods (where capture antibodies are immobilised on NPs or microfluidic channels) have been able to achieve successful CTC isolation from low mL volumes of patient blood samples with capture efficiencies spanning 65–99.9% and 72–88% respectively. Methods utilising MNPs have thus far utilised off-chip incubation of the sample (tens of minutes to a few hours) and then complete magnetic isolation on-chip. Channel-immobilised methods allow for direct injection of the sample into the antibody-modified channel. To date, as noted, the majority of platforms completing CTC isolation by antigen dependant methods have used downstream immunofluorescence imaging as the assaying method.

### 2.2 Physical isolation

Physical, or “antigen-independent” methods isolate CTCs based on differences in their physical properties from healthy blood cells (independent of antigen expression) and are thus less susceptible to issues associated with phenotypic heterogeneity, as discussed in section 2.1.^124^ Common physical differences leveraged for such methods include size, density, deformability, or charge.^125^ Size-based physical isolation techniques use filtering apparatus to (rather crudely) exploit the geometric difference in size between CTCs (mean diameter 15.6 μm), healthy red blood cells (RBCs) (7.5–8.7 μm) and WBCs (12–15 μm).^126–128^ However, a subpopulation of WBCs known as monocytes (15–18 μm) pose a significant problem.^129^ A more sophisticated method of antigen-independent CTC isolation can be achieved by microfluidic hydrodynamic separation which is achieved by judicious selection of channel geometry and can be assisted by application of an acoustic waveform.^130^ The profound variances in density and deformability of CTCs affects their loci within a channel due to enforced flow through the channel geometry. CTCs can also be physically isolated using dielectrophoresis, an approach which exploits the dielectric characteristics which differ from healthy cells. This is achieved by flowing samples through a channel under the influence of an external, non-uniform electrical field, thereby inducing directional movement of cells to facilitate isolation.^125,131^

Issues associated with these physical methods, such as size overlap, progressive membrane obstruction, and lack of tumour-specificity can result in antigen-independent isolation methods recovering a lower number of CTCs than the molecular enrichment techniques discussed earlier, and with a notably low purity (*e.g.*, 1–3%).^125,132–136^ However, their relative simplicity and straightforward integration to a microfluidic configuration justifies their inclusion as a viable CTC isolation tool.

#### 2.2.1 Physical isolation demonstrated in integrated platforms

Multiple physical isolation methods have been implemented within integrated platforms, employing size-based, dielectrophoretic, electrostatic and hydrodynamic physical isolation. Such methods have achieved isolation from volumes of 1–3 mL of whole blood.^41,54,137^

Size-based isolation on integrated devices has taken the form of either micromachined ‘hole’ or ‘trap’ arrays within the microfluidic channel system, or the use of meshes or microfilters. Mesh or microfilter approaches have achieved capture efficiencies of 87–99% from whole blood, 90% CTC purity or a 99.9% removal of WBCs.^41,137^ The simplest example of size-based filtration is presented by the use of CellSieve™ microfilters, where blood is drawn through a polymer film with an array of 7 μm diameter pores.^137^ CTCs (captured at >90% efficiency) could then be easily fixed, stained, and imaged directly on the filter. Physical isolation of CTCs has also been achieved in integrated platforms using trap channels and hole arrays, which complete a size-based cell sorting. In these reports, samples are flowed through microfluidic chips and CTCs are captured in vortices generated by chamber geometries,^138^ or by settling into hole arrays.^55,139^ These integrated methods have achieved capture efficiencies of 25–99% from a few mL of whole blood and obtained purities of 35–92%, with a WBC depletion of up to 98.7%.^55,138–140^ A recent study utilized a nanostructured array to isolate individual cells within nanodroplets, functioning as individual reactors for evaluating the secretion of matrix metalloproteinase 9 (MMP6), an enzyme crucial in the EMT process of cancer cells. The methodology involved the use of a specially designed FRET probe to assess enzyme activity without affecting cell integrity or contents, thus enabling the recovery of individual cells for subsequent analyses, as the method maintains the integrity of cells without requiring any surface modifications.^141^

Combining multiple antigen-independent isolation techniques within a chip (including those integrated with downstream analysis) has been shown to boost the achievable capture efficiency. In one report of such multiplexed isolation, Farasat *et al.* detailed a transparent (enabling CTC detection by brightfield microscopy) PDMS porous membrane, placed above microfabricated gold electrodes to achieve isolation driven by dielectrophoretic force. The PDMS microfluidic chip featured an array of 20–30 μm pores patterned by soft lithography. By applying a non-uniform electric field to the embedded electrodes, model CTCs migrated downwards and settled into the pores. This achieved isolation based on both dielectric properties and size.^142^ Unfortunately, this report did not include any detail on parameters of capture efficiency or purity.

**Fig. 6:**
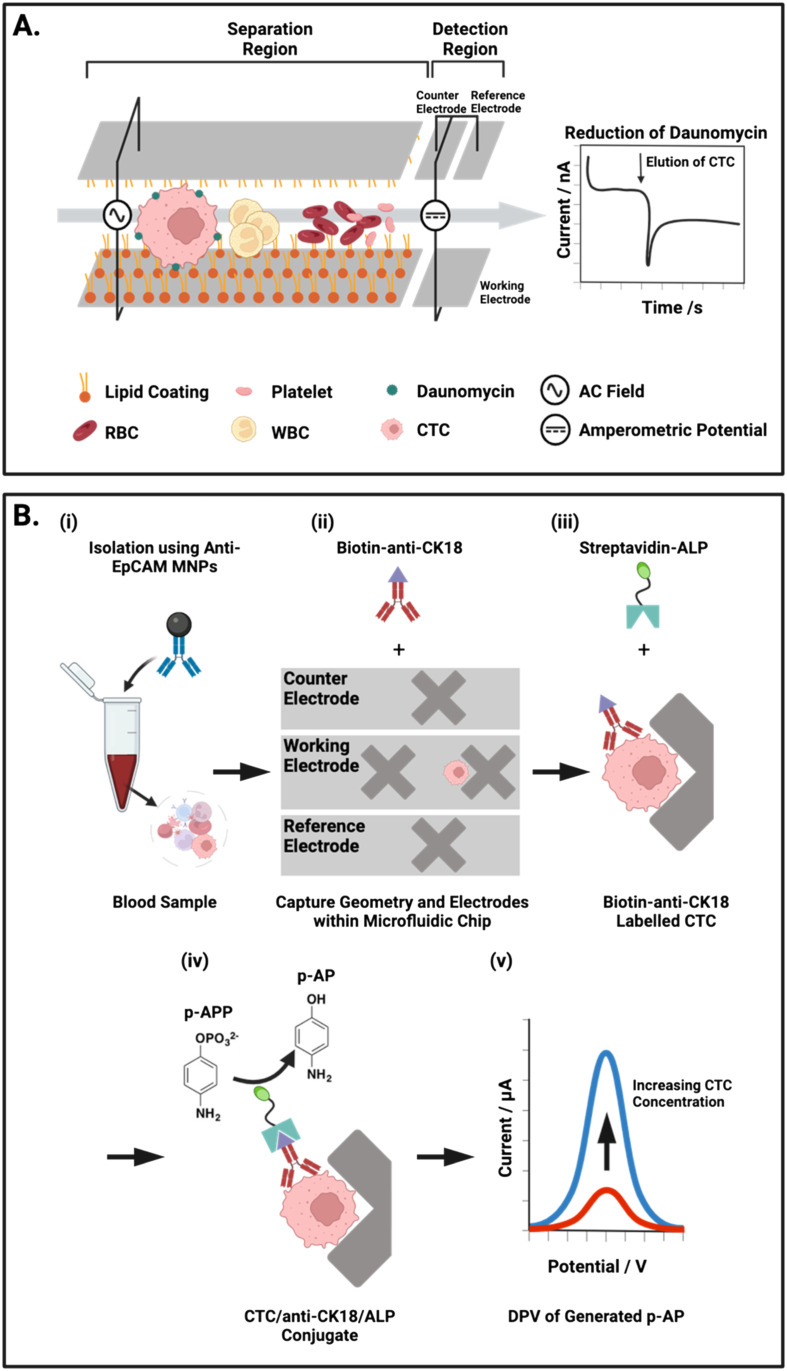
Representative examples of integrated on-chip physical CTC isolation and subsequent electrochemical detection. (A) Schematic representation of the microfluidic channel described by Gurudatt *et al.*^54^ An AC potential is applied to the carbon/polymer printed microfluidic channels to physically isolate CTCs from blood using a combination of lipid–lipid and electrostatic interactions. The reduction of daunomycin (redox-active cancer drug) labelled CTCs at the detection electrodes at the channel terminus enabled the chronoamperometric quantification of CTCs. (B) Electrochemical ELISA for the detection of CTCs from human blood. (i) Whole blood mixed with anti-EpCAM MNPs off-chip. (ii) Transfer of captured cells to the microfluidic chip where the low flow regions facilitate isolation and subsequent tagging of CTCs with biotin anti-CK18 antibodies. (iii) Labelled CTCs are exposed to a solution of alkaline phosphatase (ALP) enzyme tagged with streptavidin, forming a complex with the biotin-CK18. (iv) *p*-Aminophenyl phosphate (*p*-APP) is flowed in excess through the chip were ALP converts it to the electroactive *p*-aminophenol (*p*-AP). (v) Differential pulse voltammetry (DPV) is used to oxidise *p*-AP to generate a signal corresponding to the concentration of CTCs within the sample.

Another group have detailed two integrated approaches to separate CTCs from whole blood by selectively increasing retention time within a microfluidic channel (Fig. 6A).^54^ Firstly, an electrostatic enrichment was achieved by modifying a channel with a polymer/lipid layer. As cancer cells are known to have an increased expression of anion transporters on their surface,^143,144^ CTCs flowing through the channel were observed to have a temporary but increased electrostatic interaction with the positively charged lipid layer than healthy cells.^54^ The second design aspect capitalised on the larger size and dielectrophoretic properties of these CTCs. The application of an alternating current waveform to electrodes integrated within the walls of a microfluidic channel induced small oscillating movements of cells, perpendicular to the direction of flow (*i.e.*, towards and away from the channel walls). CTCs were shown to have slower oscillations due to their larger size, resulting in a longer retention time in the channel, thereby enabling physical separation from the smaller cellular components of whole blood. This bimodal, particle and antibody free, CTC isolation was followed by an electrochemical assay and IF imaging, both conducted directly on the chip.

**Fig. 7:**
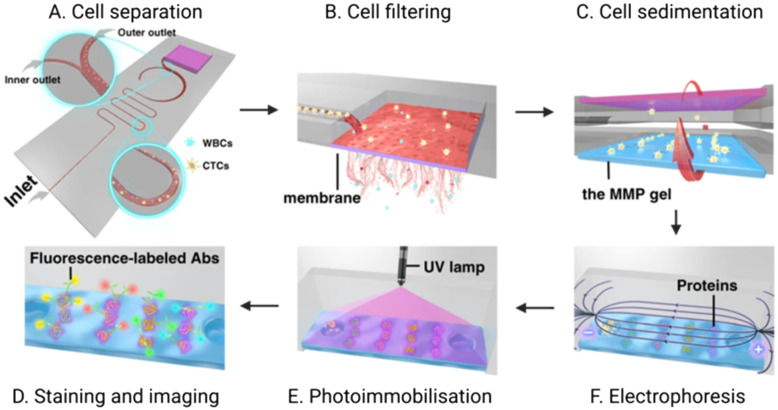
Schematic of an integrated microfluidic platform completing CTC isolation and detection. (A) CTCs are first hydrodynamically separated from WBCs; (B) the enriched CTC population is then purified *via* an *in situ* membrane filter. (C) Inversion of the chip enables CTCs to transition to the polyacrylamide gel where they are (D) chemically lysed to enable electrophoretic separation of the resultant proteins. (E) an ultraviolet (UV) light source immobilises these proteins for (F) subsequent fluorescent immunoblotting. Reproduced with permission.^41^

Another report by Abdulla *et al.* integrated both size and hydrodynamic physical isolation within a PDMS chip mounted on a glass slide. The group sought to reduce opportunities for filter blocking through the addition of a winding channel geometry to separate CTCs from whole blood prior to sample reaching a filter membrane.^41^ By adjusting the flow rate within the separation channel, they demonstrated that larger CTCs were located proximal to the channel midline, with smaller cells were pushed out distally. The relatively fast flow rate (1.4 mL min^−1^) maintained laminar flow (Reynolds number: 29.6, *cf.* <2000 is deemed laminar) and induced spatial segregation in the channel between cell types. They specifically reported the separation of white blood cells from MCF-7 cells (as a breast cancer CTC model) suspended in 0.9% saline at a purity of 70% after hydrodynamic separation alone. The enriched CTCs then continued to a filter membrane for a second stage physical exclusion before IF analysis of protein cargo by cell lysis on the chip itself (Fig. 7). Although the 70% purity obtained by this physical approach is lower than that achievable by an antigen-dependent method, achieving this degree of separation without an immunorecognition event is significant.^125,132–136^

It could be expected that technologies which combine size-based filtration to compliment antibody mediated CTC isolation should achieve an enriched CTC population with superior purity to using either one alone.^131,145^ Several proof of principle integrated platforms that demonstrate bimodal isolation strategies exist in the literature. One such report used an initial antibody-mediated bead-based capture, followed by hydrodynamic separation of the CTC–bead conjugates from the complex matrix into micromachined chip geometries with two “fences” with diameters of 9 μm and 15 μm, in which the 23 μm microspheres could be captured. The approach achieved a high capture efficiency (>85%), but a low CTC purity (20–40%) was reported, making downstream analysis more difficult.^120^

Clearly, antibody independent methods of CTC isolation warrant further investigation as recent reports which employ them in integrated isolation/detection platforms have claimed very high capture efficiencies (80–99%).^41,54,55,120,137,139,146^ Furthermore, some reports herein discussed have combined antigen-dependent and physical means of isolation (*i.e.*, antibody-functionalized chip with inertial microflow^147^ or antibody-functionalised nanoparticles injected into a microfluidic chip^148^), achieving high capture efficiencies of 90–94%. However, sample input across these works has not been consistent, with some groups completing their enrichment on unmodified patient samples,^137,139^ whilst other studies utilised pre-processing, including RBC lysis, various washing steps,^41^ and PBS dilution of blood.^55,138^ As with integrated platforms applying antigen-dependent isolation, the vast majority of physical methods utilised within integrated formats have been paired with optical detection methods,^41,55,137–140,142,149^ with only two reports of electrochemical means of detection.^54,146^

## 3 Detection of CTCs

Once CTCs have been isolated on-chip using the antibody-mediated or physical methods discussed in sections 2.1 and 2.2, subsequent detection is required (*i.e.*, quantitation and/or identification). As mentioned in the previous examples, this can involve either quantification (*i.e.*, enumeration), or optical or physical confirmation that captured cells are CTCs. Methods available can be broadly categorised, then, as those which are optical, electrochemical or physical.

Optical methods are most commonly fulfilled through well-established immunofluorescence (IF) imaging, using multichannel fluorescence staining of cellular markers.^55,56,115,116,119,121,122,137,139,140,149^ CTCs are typically detected based on CK (cytokeratin)^positive^, EpCAM^positive^, nuclear DAPI^positive^, and CD45^negative^ signatures.^69^ However, IF requires cell fixation, numerous manual transfer steps and ultimately, interpretation by a clinical cytologist or pathologist. The laborious and time consuming (typically ∼5 active working hours) nature of the method, may prohibit the translation of IF-based CTC screening platforms into routine clinical practice. Additionally, relying on this approach reduces achievable throughput, introduces multiple opportunities for systematic or random errors, and ultimately presents an increased cost per test.^20,150^

Furthermore, a perfectly specific positive marker for CTC identification has not been identified, as EpCAM can be, as noted earlier, downregulated during the EMT and CK is not universally expressed across all tumours. This results in CK^negative^ and EpCAM^negative^ CTCs evading detection when relying on these markers. This is concerning as CTCs with this signature have already been identified in peripheral blood of patients with breast cancer.^93^

The enzyme-linked immunosorbent assay (ELISA) workflow, typically used for high-throughput protein biomarker assays, is another optical technique applicable to CTC detection, whereby primary and secondary antibodies can be used to first capture, and then generate a quantifying signal. This format can be adapted to produce an electrochemical output by exchanging the optically responsive label/substrate for a redox-active species produced by the enzyme-conjugated secondary antibody. Using voltammetry or amperometry to assay the production of such, the CTC concentration in the sample can be inferred. Label-free electrochemical methods are also applicable to CTC detection in integrated platforms as techniques. This includes electrochemical impedance spectroscopy (EIS) which enables the direct assessment of an interaction between CTCs and an electrode surface. Electrochemical diagnostics are already known to have sufficient sensitivity to assay metabolites and biomarkers in human samples (glucose, lactate, pH, pO_2_ and pCO_2_).^112,151–155^

Additionally, the cost and footprint of electrochemical instruments has been decreasing over the last decade, with a 10-gram potentiostat recently demonstrating similar results in amperometric virus detection, for example, when compared to an instrument 50 times its size, with no statistically significant difference in the signal obtained (*p* < 0.05).^156^ A miniaturised electrochemical means of CTC analysis integrated with on-chip isolation presents a real opportunity for an automated (or semi-automated) configuration with a high potential for clinical impact.

Detection can also be achieved by using physical differences between CTCs and other cell populations.^94,157^ These do not employ immunorecognition agents or staining protocols and therefore have a rapid TAT when compared to methods that do (*i.e.*, immediate detection with high-speed camera or pressure gauge).^138,158^ Physical methods of CTC detection have been less frequently reported (integrated or otherwise), but one non-integrated example utilised specialised software to confirm CTC identity following isolation with CellSearch® based on size, nuclear–cytoplasmic ratio, and elongation (*i.e.*, cell morphology).^94^ These technologies have not yet been widely adopted, however a few examples of physical CTC detection on integrated platforms are discussed below (section 3.2.3).

### 3.1 Integrated on-chip CTC detection

**Fig. 8:**
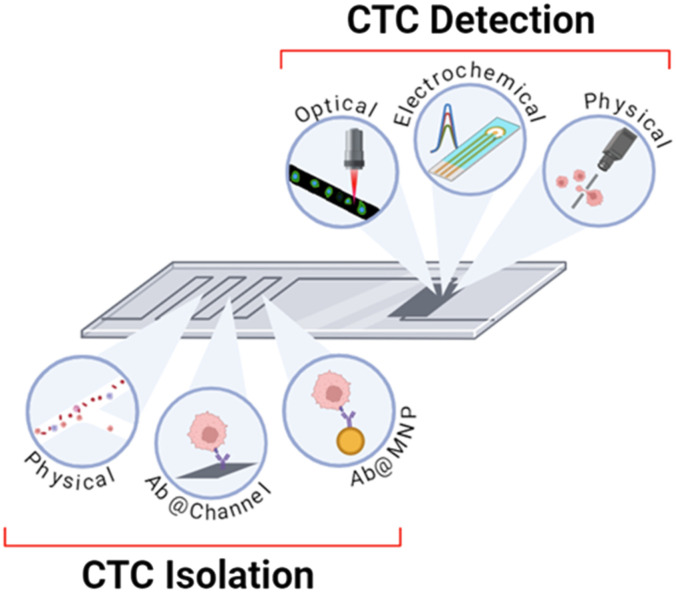
Schematic summary of the methods of isolation and detection that are utilised within integrated platforms discussed herein. Isolation can be completed using physical methods, antibody-modified channels, and antibody-modified nanoparticles. Detection can be achieved through optical, electrochemical, or physical methodologies.

Optical, electrochemical and physical methods of CTC detection have been employed in integrated on-chip platforms where the channels have been patterned in a PDMS substrate or otherwise microfabricated by lithography. The detection methods are summarised in Table 1 and Fig. 8 and the sections below provide a description of their operational principles and methods of integration.

### 3.2 On-chip integration of optical CTC detection

As briefly mentioned in section 2, optical CTC detection is often utilised within integrated on-chip platforms, perhaps the simplest being direct brightfield or scanning electron microscopy (SEM) of the chip region in which cells were isolated.^137,142^ Such methods have been performed by simply placing the microfluidic chip under a microscope. However, these basic methods offer little information on the captured CTCs, and no prior works have reported a detection limit.

IF staining is by far the most prevalent technique employed thus far for on-chip CTC detection (Table 1), but other available optical techniques include up-conversion luminescence (UCL) imaging and immunoblotting. Researchers frequently default to IF staining, despite the above noted limitations, due to the well-established protocols and literature-supported staining signatures.^69^ IF within integrated platforms is performed by fixing cells within the channels, flowing staining reagents through, and conducting fluorescence imaging of the chip. For this reason, IF is ubiquitously used to validate CTC detection on platforms where the primary assay was completed by other means (*e.g.*, electrochemical impedance spectroscopy (EIS)^112^ or deformability cytometry^138^).

The majority of integrated platforms detect CTCs based on a DAPI^positive^, CK^positive^, and CD45^negative^ signature^56,115,139^ with numerous teams including EpCAM positivity in their CTC definition.^55,137,140,149^ Some reports have enhanced the specificity of their assays towards metastases of distinct origin by including markers beyond the typical selection described above. In one example of this, Lee *et al.*, conducted IF staining of the oestrogen receptor (OR) and progesterone receptor (PR) with an aim to discriminate OR and PR positive CTC subtypes; highly relevant in breast cancer diagnoses (Fig. 5B).^122^ Similarly, Shi *et al.* included single cell staining for HER2 on their valve controlled PDMS microfluidic chip. CTCs were shuttled through each region of the device, and subsequently stained, washed and blocked before the entire chip was imaged beneath an inverted microscope, an approach with minimal opportunity for cell loss or damage.^121^

Of the optical integrated methods, CTC detection by IF has been shown to achieve some of the most impressive detection limits ranging from 1 cell/1.5 mL (ref. 139) to 10 cells/1 mL in whole blood.^149^ However, gains are being made in lowering this value in an attempt to sample smaller blood volumes using alternative optical techniques such as isothermal nucleic acid detection and immunoblotting.

One unique report of this by Su and team detailed the use of rapid isothermal nucleic detection assay (RIDA) for CTC detection following upstream isolation by antibody-functionalised microbeads and hydrodynamic separation.^120^ Detection was achieved using biotinylated oligo-DNA conjugated to an anti-EpCAM antibody *via* streptavidin. This difunctional complex enabled both high specificity capture and sensitive PCR amplified optical detection of the fluorescent signal attribute to the DNA–antibody conjugates. This approach is reported to be effective enough to support a detection limit of 50 cells/1 mL of whole blood.

Another unique report of optical detection in an integrated platform described by Abdulla *et al.* utilised an immune-affinity technique *via* immunoblotting to study protein expression within captured CTCs.^41^ In this work, the team conducted single-cell western blot analysis of captured CTCs by inverting the PDMS chip to transfer the captured cells into microwells in a standard electrophoresis gel (Fig. 7). The cells were lysed *in situ*, enabling single cell Western blot using primary and secondary antibodies. This method achieved a detection limit of 23 cells/2 mL whole blood, a capture efficiency exceeding 98%, and purity of 90%. Importantly, this work demonstrated an ability to analyse protein expression on the single-cell level, allowing for profiling of CTCs (not just detection) beyond the cell surface markers or physical attributes revealed by IF.

#### 3.2.1 Spectroscopic means of integrated on-chip CTC detection

Detection of CTCs has been achieved using spectroscopic means in two integrated platforms to date.^113,118^ The use of nanoparticles (NPs) has already been discussed above as a toolbox for enriching CTCs from complex media (section 2.2); in this section we highlight how their unique physical properties support spectral CTC detection.

In one example, Wang *et al.* first captured CTCs using antibody functionalised MNPs, which could be manipulated and detected on chip.^118^ The cell–particle conjugates proceeded to a silicon nanowire substrate (placed above a permanent magnet) within a PDMS microfluidic channel. The immobilised MNPs/CTC complexes were then quantified using up-conversion luminescence (UCL) by placing the entire chip beneath a 980 nm laser source, allowing for immediate visualisation of CTCs on the chip. This method required only 5 mL of blood from patient samples, demonstrated a detection limit of 10 cells/2 mL of PBS, and a release efficiency of ∼80% with *in vitro* culture of released cells for 7 days following.

**Fig. 9:**
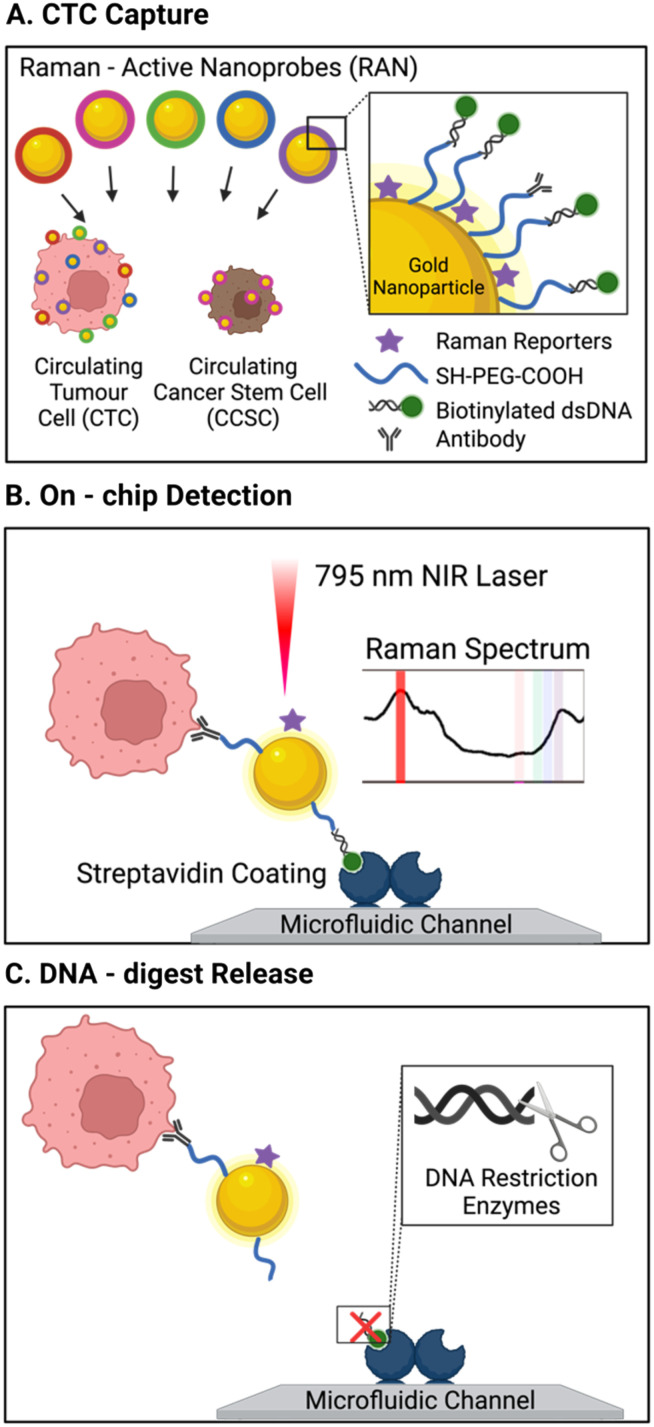
Schematic of a platform with integrated isolation of CTCs *via* antibody-conjugated Raman-active nanoprobes (Au-NP) wand Raman spectral detection. (A) CTCs were immuno-labelled with 5 types of surface antibody/Raman reporter functionalised AuNP. (B) Biotin units on the capture particles enabled CTC isolation on the streptavidin modified channels, where Raman spectral intensities revealed the degree surface marker expression. (C) CTC release was then accomplished by the addition of a restriction enzyme to cleave dsDNA AuNP linker. Reproduced with permission.^113^

Immunofluorescent staining of on-chip isolated CTCs provides a viable approach for CTC isolation and detection in microfluidic platforms, akin to methods applied in traditional protein immunohistochemistry. This approach has been applied to the label-free isolation of CTCs from ascites and peritoneal lavages using a specially designed continuous flow, size-based, cell trapping array-chip followed by immunofluorescence mapping of EpCAM, YAP-1, HER-2, CD45 to identify CTCs down to a single cell.^159^ Despite the well-established nature of immunofluorescence-based assays, Cho *et al.* have, for example, acknowledged a challenge in mapping multiple cell surface markers because of fluorophore spectral overlap and associated difficulty in interpretation.^113^ Thus, the researchers developed 5 antibody-functionalised gold nanoparticles (Raman-active nanoprobes/RANs), to isolate and tag CTCs from whole blood. The probes were functionalised towards HER2, CD133, EGFR, EpCAM and MUC1, with each RAN having a known spectral fingerprint. By examining the CTC enriched sample with surface enhanced Raman spectroscopy (SERS), the team used the RANs characteristic peaks to identify and quantify circulating cancer stem cells (CCSC), a rare sub-type of CTCs (Fig. 9). SERS mapping imaging was conducted directly on the chip itself using an inverted optical microscope. This allowed for on-chip, multiplexed phenotypic analysis of the captured CTCs based on their spectral identities. This configuration was shown to predict metastasis in a xenograft model with an exact correlation between CCSC concentration and prevalence of liver metastasis. In a recent development, a comparable methodology employed three distinct subtypes of Ab-functionalized Raman-active AuNPs to characterize the heterogeneity of surface protein biomarkers on-chip isolated CTCs. The configuration used on-chip under alternating current-induced mixing of the cells close to chip-laden electrodes, improving CTC capture efficiency, while minimizing non-specific adsorption of loosely bound species within an Ab-functionalized microfluidic chip.^160^ This configuration was capable of stratifying CTCs and identifying drug-resistant forms by mapping cancer-specific cell-surface biomarkers. Another approach for mapping CTC surface biomarkers through after a semi-automated single step isolation of CTCs on a micropore membrane filter coupled to single cell Raman mapping was proposed.^161^ In this approach, isolated cells were labelled with SERS-active AuNPs functionalized with specifically engineered aptamers capable of distinguishing isolated CTCs from residual WBCs. While this set-up did not incorporate microfluidic control, it has potential to serve as a readily accessible single platform for such applications.

#### 3.2.2 Electrochemical means of integrated on-chip CTC detection

As noted previously, electrochemical platforms are progressively decreasing in size and cost and are readily integrated within microfluidic configurations. This enables the assembly of a complete testing platform in a small footprint, with only a small working area needed to place a 2 or 3 electrode sensor into a microfluidic device, particularly if microelectrodes are utilised.^162^

As described earlier, the predominant means of CTC isolation prior to on-chip electrochemical assays are Ab-functionalised channels.^112,114^ An exception to this, is the work of Safaei *et al.* where CTCs were first enriched in whole blood by anti-EpCAM modified MNPs and subsequently assayed for on-chip using differential pulse voltammetry (DPV), this is an exemplar of an electrochemical ELISA arrangement. In DPV, the current response associated with the voltage pulse is recorded and analysed to examine the faradaic interactions at the electrode surface. The researchers integrated working, counter and reference electrodes to their microfluidic platform by lithographic patterning of 50 nm gold on a glass substrate, and these tracks were subsequently passivated to create isolated electrode apertures. Microfluidic channels were then constructed above the electrodes using photolithography and subsequently capped with a PDMS layer. To obtain a DPV signal, Safaei *et al.* immobilised and fixed (using 4% paraformaldehyde) their MNP enriched CTCs on ‘X’ shaped geometries within the chip. Prior to this, these conjugates were tagged with biotinylated anti-CK and then exposed to a solution of streptavidin/alkaline phosphatase (ALP) (Fig. 6B).^117^ Subsequent DPV scans generate substrate dependent amperometric currents that report directly on CTC concentration within the sample. Although this assay involved the numerous incubation steps characteristic of a typical microtiter plate ELISA, the overall protocol complexity was reduced. This is chiefly due to the fabricated microfluidic chip which harboured MNP enriched CTCs for the duration of the ELISA protocol. By simply exchanging the running solution, CTC isolation and detection was completed on-chip in a facile manner.

The assay achieved a detection limit of 2 cells per mL of whole blood, requiring only 1 mL to complete the analysis. Additionally, the researchers reported a capture efficiency of 85% and, due to the MNP separation, low contamination from WBCs at ∼100 cells in 1.5 μL buffer. The electrochemical assay time was not reported; however, each DPV measurement required around 1 minute per sample, a notable contrast to the multiple hours typically required for the IF methods discussed in section 3.1.

In a further example of a fully integrated voltammetric CTC assay, researchers assembled a simple, single channel PDMS microfluidic chip using soft lithography above a screen-printed electrode (SPE). The latter was functionalised with polymer and anti-MC1R antibody to capture cells *via* immune-affinity interactions.^114^ The group showed that an increasing CTC (SK-MEL-2) concentration suspended in a redox active running buffer resulted in a log linear decrease (over 10–9000 cells per mL) in voltammetric peak amplitude due to the blocking effects of surface captured cells. In an attempt to reduce non-specific binding from potentially interfering proteins present in real samples, the Ab-modified SPEs were further incubated with bovine serum albumin (BSA). The performance of the sensor was expected to diminish in progressively complex media, and indeed a 20% signal loss was observed over a 3-hour experiment in spiked buffer. In whole blood or other complex media, this degradation is almost certainly substantially higher. The myriad of small cells and proteins present in a human sample are a known issue for electrochemical biosensors as these can readily foul the electrode surface, erroneously reducing the signal from a solution phase redox probe (*i.e.*, poor signal-to-noise ratio).

This can, of course, be remediated by a greater consideration of electrode surface engineering, or integration of this detection method with any of the antigen dependant or physical methods of CTC isolation discussed section 2. A porous 3D channelled PDMS architecture coated with Ab-functionalized conductive polymer was proposed as a novel platform to first, capture EpCAM^positive^ CTCs, then detecting isolated CTCs leveraging the preferential adsorption of platelets onto the CTCs' surface. The adsorbed platelets activate the catalytic decomposition of H_2_O_2_ mediated by specifically designed metalloporphyrin (cobalt hematoporphyrin).^163^ While this study demonstrates a novel signal generation strategy in identifying patients at risk of hematogenous metastasis (as quantified by the increase in the number of platelet-interacted CTCs), it does not make any reference to the impact of background noise (due to nonspecific adsorption of platelets to the electrode surface) or explore the detection limits.

Chronoamperometric methods, where current is recorded as a function of time, can be employed within microfabricated chips to obtain transient data as CTCs flow through a channel as an alternative to an endpoint assay. This approach has been employed by researchers to quantify CTCs through their association with redox active daunomycin (DM), a potent anti-cancer drug.^54^ DM was shown to specifically adsorb to a model CTC (HeLa) with low response to a non-cancerous control (HEK-293). A spike in the oxidation current recorded by the electrodes at the channel terminus indicated the presence of a DM labelled CTC eluted from the channel. Due to the upstream physical CTC isolation strategy discussed in section 2.2.1, the time lapsed from the start of sample flow could be used to estimate CTC size, aiding in discrimination between any erroneously labelled healthy cells and, additionally, differentiate between multiple species of CTC. This is an interesting, direct faradaic alternative of the cell tagging immunogenic strategies described earlier. The entire protocol was successfully integrated into a microfluidic chip where the channels and electrodes were formed by screen-printing carbon ink between two glass slides, these then used to probe the oxidation of CTC-tethered DM (Fig. 6A). Importantly, in analysing a significant number of cancer patient samples, with 1 mL volumes incubated off-chip with the drug (∼45 minutes), prior to isolation and detection on-chip, detection limits were reported at 7 cells per mL.

Electrochemical impedance spectroscopy (EIS) involves the application of an AC potential between electrodes and records the resultant current, with respect to frequency or time. This method enables researchers to discriminate between electrode interactions occurring at characteristic frequencies, *e.g.*, simultaneously probing diffusion and charging of the electrochemical double layer and is exquisitely responsive to capture events at a suitably modified electrode. In one report, model CTCs (MCF-7) suspended in PBS at 100 cells per mL were flowed through a simple PDMS channel, captured at the electrode surface *via* sensor functionalisation with anti-EpCAM and anti-CD36 antibodies, and quantified through the associated change in charge transfer resistance (*R*_ct_).^112^ The researchers reported the quantification of 3 MCF-7 breast cancer cells as CTC analogues in buffer and claim to detect the presence of CTCs in 1 mL of canine cancer patient blood, although no downstream analysis or recovery experiments were reported.

In another example of integrated EIS quantification, CTCs were isolated *via* pre-concentration onto the electrode surface by application of a dielectrophoretic (DEP) current. This approach offered a rapid TAT, with pre-concentration of CTCs by DEP in 2 minutes, and EIS quantification within 1–2 minutes.^146^ Similar to the DPV CTC detection method discussed earlier, these sensors are subject to reduced performance in blood due to unwanted electrode fouling. The analyses here were thus completed in lysed blood (requiring pre-processing for RBC lysis) or sucrose buffer, respectively, to present CTCs in a low complexity buffer to the functionalised electrodes.

In summary, electrochemical methods of detection have been shown to be both easy to integrate, and analytically powerful. The typical three electrode configuration has been implemented by directly sealing SPEs beneath a PDMS chip or using tracks of conductive carbon ink as both the electrodes and the walls of microfluidic channels. These rapid assays do not present the notably high costs and TAT presented by IF and have been shown to support an outstanding performance, with detection limits of 2–7 cells per mL in only 1–2 mL of whole blood.^54,112,114,117,119,146^

#### 3.2.3 Physical means of integrated CTC detection

While the physical attributes of CTCs are often exploited for isolation (section 3) integrated platforms have also utilised this approach in detection. The two most notable reports use pressure variance measurement and deformability cytometry. One such proof of principle device reported by Namli *et al.* used a gauge attached to a 6 mm microfluidic chip (on silicon wafer substrate) to measure the pressure required to induce hydrodynamic cavitation in a sample matrix. This was shown to be reduced when CTCs were present, despite the presence of Jurkat cells as models for healthy WBCs.^158^ Pressure variation was explained by the heterogeneous nucleation theorem, whereby cells act as stream nucleation sites for the generation of bubbles in the liquid sample. While this offers a unique method of physical CTC detection, no metrics of platform performance (*i.e.*, capture efficiency or purity) were published other than the detection limit. Other particulate matter could similarly reduce the cavitation pressure, meaning that specificity was likely poor. Thus, there is room for further development of such an approach.

In a second example of a purely physical assay, Che *et al.* utilised deformability cytometry where CTCs were shown to deform to a greater extent than other healthy blood cells when introduced to a microfluidic channel that induced hydrodynamic stretching.^138^ This was analysed using a high-speed camera, focussed on this deformation channel, synchronised to the release of cells from reservoirs in which they were isolated. The team used additional off-chip IF for validation of detection following CTC elution, using a CK^positive^/CD45^negative^/DAPI^positive^ signature. Importantly, it was noted that deformability cytometry successfully detected CTCs in 94% of non-small cell lung cancer patients, whereas IF reported only 71% of such samples to be CTC positive.

Notably, these examples of physical detection, though underdeveloped, are completely antigen-independent. However, their sensitivity (detection limit of 50 cells per mL in media supplemented with Jurkat cells and 300 cells in 10× diluted blood, respectively) is markedly poorer than that reported to be associated with other methods discussed. Additionally, they both require pre-processing of blood (40 minutes for RBC lysis and removal and 10 times dilution in PBS, respectively). Despite these limits, these are low-cost approaches, simple to operate, and enable rapid CTC analysis on biomarker independent platforms.^158^

## 4 Conclusion

CTC analysis can provide crucial insight into cancer metastasis and tumour evolution.^47^ At present, methods for CTC isolation and detection have been predominantly built to separate isolation (CTC capture) and detection (verification of captured cell identity or quantification of captured cells).^47^ The multistep nature of the assay increases time, cost, and manual labour required, and introduces cell loss and significant inter-test variability.^2–5^ The integration of CTC isolation and detection onto a single microfluidic chip promises to decrease time, cost, and variability of both enrichment and analysis. The use of such configurations has thus far supported capture efficiencies as high as 99% (ref. 122) and detection limits as low as 2 cells per mL whole blood (Table 2).^117^

**Table 2:** Summary of key parameters and details of integrated platforms discussed within this review. “—” indicates that the given parameter is not detailed within the given report. “N/A” indicates that the given parameter is not relevant to the given report

Target cells	Isolation method	Detection method	Detection limit	Flow rate	Blood pre-processing; duration	Volume	Capture efficiency	Purity	Other notes	Surface antigen targeted	Ref.
MDA-MB-231	Variance in pressure required to initiate cavitation	High speed camera and pressure gauge	50 cells per mL culture medium suppl. with Jurkat cells	—	>40 minutes for RBC lysis; detection “within minutes”	—	—	—	—	N/A	158
- Breast cancer											
H1975	Dislocation herringbone μF geometry, immobilised anti-EpCAM Ab	IF (CK 18+/CD45−)	5 cells per mL whole blood	20 μL min^−1^ for cells spiked in blood optimized flow rate	No blood pre-processing; 5 hours per sample	—	H1975 87%, A549 78%, H460 72%	99.6%	—	EpCAM	116
A549											
H460											
- Lung cancer											
A549	Off-chip incubation with anti-EpCAM MNPs, μF on-chip magnetic capture	UCL imaging	10 cells/2 mL PBS	1 mL h^−1^ optimized flow rate	RBC lysis and 2 h incubation with anti-EpCAM MNPs; not detailed	0.5 mL spiked whole blood, 5 mL patient blood sample	>80%	—	80% release efficiency; released cells could be cultured for 7 days	EpCAM	118
- Lung cancer											
VcaP	Immuno-modified MNPs, hydrodynamic separation on chip	EChem oxidation of redox-active secondary Ab, IF validation	2 cells per mL whole blood	—	20 min incubation with anti-EpCAM MNPs; ∼3 h to pump sample through, signal read out 1 min per sample, ∼1.5 h for IF	—	85%	Average capture of ∼80 WBCs	—	EpCAM	117
- Prostate cancer											
MCF-7	Hydrodynamic μF cell sorter with size exclusion membrane filter	Single-cell western blotting	23 cells/2 mL whole blood	1.4 mL min^−1^	10 min RBC lysis, 15 min washing; not detailed	7.5 mL blood sample	87–98.5%	89.92%	Analysis of EMT in CTCs isolated from patient samples	N/A	41
- Breast cancer											
A459											
- Lung cancer											
HeLa											
-Cervical cancer											
MCF-7	Antibody functionalised electrodes	EIS, IF validation	100 cells per mL PBS	—	RBC lysis; not detailed	—	—	—	—	EpCAM, CD36	112
- Breast cancer											
SK-MEL-2	Abs on PANI modified carbon SPEs	EChem reduction of solution redox probe	1 cell/1 mL PBS suppl. with human cells	1.5 mL min^−1^	Not detailed; not detailed	—	—	—	—	MCR1	114
- Skin cancer											
A549	Off-chip incubation with daunomycin, AC field isolation on μF chip	EChem oxidation of daunomycin	7 cells per mL whole blood	5 μL min^−1^ for 5 minutes followed by 2.5 μL min^−1^	40 min (30 min incubation with daunomycin, 5 min centrifuge); not detailed	1 mL of whole blood	Average of 95+–1.5%	92+–0.5%	—	N/A	54
- Lung cancer											
MDA-MB-231											
- Breast cancer											
HeLa											
-Cervical cancer											
MCF-7	Off-chip incubation with immuno-modified magnetic beads, on-chip application of magnetic field	IF (CD45−/HER2+/Hoechst 33 342+)	10 cells/2 mL whole blood	Optimal flow rate 90 μL min^−1^ for spiking and 1 μL min^−1^ for patient samples	Dilution of whole blood, incubation with magnetic beads for unknown time; 240 min for entire process	—	93%	—	On-chip cell lysis for subsequent RNA-seq	EpCAM	121
- Breast cancer											
H446	Size based sorting in trap channel based on flow resistance	IF (CD45−/CK+/DAPI+), additional IF of EpCAM and vimentin	5 cells per suspension of 100 000 WBCS	Optimized injection pressure of 10 mbar	Ficoll-Paque processing; not detailed	—	92–99% depending on WBC count	<80–92% depending on WBC concentration	Achieved release efficiency of >97%	N/A	140
- Small cell lung cancer											
H1975											
- Non-small cell lung cancer											
MCF-7	Size-selective vortex trapping with chip geometry	Deformability cytometry (DC), IF for validation (CK+/CD45−/DAPI+)	300 cells per unknown volume of 10× diluted blood	8 mL min^−1^	10× dilution of blood in PBS; not detailed	—	25–35%	35.1+–7.3%	98.7+–1.5% of cells transfer from capture to assay region of chip	N/A	138
- Breast cancer											
MCF-7	1) Off-chip incubation with anti-EpCAM microbeads	Signal amplification using DNA–Ab conjugates in RIDA	50 cells/1 mL of whole blood	Optimal flow rate of 1–3 mL h^−1^, 3 mL h^−1^ used for patient samples	25-Minute centrifugation for WBC separation; not detailed	—	>85%	20–40%	Captured cells proliferated normally in culture	EpCAM	120
- Breast cancer											
BxPC-3											
-Pancreatic cancer											
HeLa	2) Inertial micro-flow system										
- Cervical cancer											
PANC-1											
-Pancreatic cancer											
PC3	Dielectrophoresis on-chip	Brightfield microscopy	Not available	Optimal flow rate of 3 μL min^−1^	Not detailed; not detailed	—	—	—	DEP buffer can have effects on cells' gene expression (>15 min)	N/A	142
- Prostate cancer											
MCF-7	Off-chip incubation with Ab-conjugated MNPs, magnetic ranking on-chip (MagRC chip)	IF (DAPI+/CK+/CD45−)	10 cells/1 mL unprocessed blood	600 μL h^−1^	30 minute incubation with anti-EpCAM nanobeads; not detailed	1 mL of blood used for patient samples	SKBR3 97+–3%, PC-3 90+–2%, MDA-MB-231 90+–3%	—	92% recovery, 98% viable	EpCAM, (plus HER2 and N-cadherin for SKBR3 cells)	115
SKBR3											
- Breast cancer											
PC3											
- Prostate cancer											
MDA-MB-231											
- Breast cancer (mesenchymal properties)											
MDA-MB-231	Off-chip incubation with anti-HER2 MNPs, magnetic ranking on-chip	IF of estrogen receptor (ER) and progesterone receptor (PR)	—	3 mL h^−1^	No experiments with patient samples conducted	No experiments with patient samples conducted	MDA-MB-231 65.3+–1.6%, BT-474 99.3+–0.4%, SK-BR-3 99.9+–0.1%, MCF-7 92.9+–1.8%	—	—	HER2	122
BT-474											
SK-BR-3											
MCF-7											
- Breast cancer											
MCF-7	Size based isolation with CellSieve microfilter	IF (CK8/18/19+, EpCAM+, DAPI+), H&E staining	—	5 mL min^−1^ for cells spiked in whole blood	No blood pre-processing; not detailed	—	Approx. 90%	>99.9% removal of WBCs from blood sample	Cell lines adhered and grew on the microfilters, 97+–2% release efficiency of MCF-7 cells	N/A	137
MDA-MB-231											
SK-BR-3											
- Breast cancer											
PANC-1											
- Pancreatic cancer											
A549	Size based isolation with PDMS membrane filter-based microdevice	IF (CK-PE+/CD45-FITC−/DAPI+)	1 cell/1.5 mL of whole blood	Optimized flow rate of 10 mL h^−1^ for cells spiked in whole blood	No blood pre-processing; not detailed	—	>92% for A549 and SK-MES-1 cells; 99% for H446	—	—	N/A	139
SK-MES-1											
H446											
- Lung cancer											
PC-3	ODEP following IF identification of cells of interest	IF (CD45−/Hoechst+/EpCAM+)	10 cells per mL whole blood	Optimized flow rate of 2.5 μL min^−1^ for cells spiked in whole blood	No blood pre-processing; not detailed	8 mL of blood for spiking experiments	41.5%	As high as 100%	Detection occurs prior to isolation	N/A	149
- Prostate cancer											
SKBR3	SMART-chip; microchannels with immobilized anti-EpCAM Ab	Impedance counting and viability of unlabelled single cells; IF (DAPI+/CD45−/Pan-CK+)	—	—	No blood pre-processing; 3.5 h	2 mL of blood processed for patient samples	>80%; 73% for anti-EpCAM PC linker with SKBR3 cells in whole blood	>85%	Quick (2 min) and efficient (>90%) release of affinity-captured CTCs *via* photo release with visible blue light	EpCAM	119
- Breast cancer											
MCF-7	3D PDMS scaffold with immobilized anti-EpCAM Ab	IF staining (DAPI+/CK+/CD45−)	10 cells/1 mL whole blood	Optimized flow rate of 100 μL min^−1^ for cells spiked in whole blood	No blood pre-processing; ∼4.5 h	1 mL of whole blood for spiking experiments	Average capture efficiency of 88.4%	—	CTC clusters were also detected and imaged in patient samples	EpCAM	56
- Breast cancer											
MCF-7	Raman active nanoprobes (RANs) conjugated to Ab (anti-CD133, anti-EpCAM, anti-HER2, anti-MUC1) with biotinylated dsDNA for isolation	*In situ* subtyping by Raman barcoding system	—	10 μL min^−1^	2× dilution with PBS, Ficoll-Paque processing, centrifugation for WBC exclusion, 30 min incubation with RANs; not detailed	4 mL blood for spiking experiments	90%	—	Detects circulating cancer stem cells (CCSCs) as well as CTCs, ability to release captured cells using restriction enzymes	CD133, EpCAM, HER2, MUC1	113
MDA-MB-231											
SK-BR-3											
- Breast cancer											
A549	Windmill-like hole array for size-based isolation	IF staining (DAPI+/CD45−/Ep-CAM+ CTC signature)	<10 cells per mL blood	2 mL min^−1^	Unspecified pre-treatment and dilution with 10 mL PBS; not detailed	1–3 mL whole blood for patient samples	93% for A549 cells; 90% for HeLa cells	WBC depletion rate of 98.7%	—	N/A	55
- Lung cancer											
HeLa											
- Cervical cancer											
A549	DEP	EIS	3 cells	Flow rate of 1.2 μL min^−1^	Not detailed; electrical operation ∼10 min	—	Approx. 80%	—	Detection limit achieved at 50 kHz	N/A	146
- Lung cancer											
A549	SDAM	FRET (peptide probe for MMP9 enzyme activity)	Single cell	—	Diluted 10 mL blood (blood : PBS = 1 : 1)	5 mL	—	—	Evaluate EMT and cancer metastasis	MMP6 enzyme activity	141
- Lung cancer											
HGC-27	IMD composed of a spiral chip (using the DFF technique) and a DLD chip	IF staining (EpCAM, YAP1, HER-2, CD45)	1 cell/10 000 cells	Optimized flow rate of 12 μL min^−1^	Ascites and peritoneal lavages	N/A	63%	73%		EpCAM	159
- Gastric cancer											
SK-MEL28	ac-EHD with antibody modified chip	Raman signature for MCSP, MCAM, ErbB3, and LNGFR (melanoma) and PD-L1 and EGFR (lung cancer)	—	Loaded immediately into the chip with ac-EHD applied for 10 min	PBMCs suspended in 2.0 mL RPMI 2 h	—	60%	N/A		MCSP for melanoma cells	160
- Melanoma											
NSCLC										EGFR for lung cancer cells	
- Lung cancer											
A549	Microporous parylene membrane	Raman intensity	N/A	Dropped onto the micropore membrane for filtration	A549 cells in 100 μL culture medium into 400 μL blood	—	91%	90%	Aptamer probe selected for preferential binding to CTCs	Size based filtration	161
- Lung cancer											
SK-BR-3	3D electrode integrated within a microfluidic flow channel	SWV	—	40 μL min^−1^	Not discussed	—	71%	N/A		EPCAM	163
- Breast cancer											

The majority of CTC isolation methods in integrated platforms rely on immuno-affinity,^112,114,115,118,120,122^ but others, based on cell deformability,^138^ inertia force,^41^ and size variance^158^ have been documented. Integrated detection methods range from direct imaging of a transparent PDMS chip, often employing some derivative of immunofluorescent staining, to electrochemical detection using electrodes embedded within the platform.^54,112,114,117,119,146^

Antigen-dependent methods of isolation used in integrated systems have achieved capture efficiencies of 80–97%,^56,112–122^ and purities of up to 99.9%.^56,119,122^ Physical methods of isolation completed on a single chip are reportedly capable of obtaining CTCs with capture efficiencies of 25–99% (ref. 41, 54, 55, 120, 137–139, 146) and associated purities spanning 20–92%.^54,120,138^ While the purity metric is typically lower with physical means of isolation, their utility should not be underestimated as they allow for the recovery of label-free CTCs, delivering an ideal starting point for interference-free downstream analysis. CTC recovery following antigen-dependent isolation has been also reported with release efficiencies ranging from 80–97%,^115,118,119^ with one report even demonstrating a post-assay proliferation of isolated cells in culture.^120^ Such downstream release and culture represents an advantage for physical means of isolation, although antigen-dependent isolation methods are also able to achieve release if appropriately designed.

Looking beyond isolation, the platforms discussed in this review have achieved CTC detection from low volumes (down to 1 mL whole blood),^54,120^ with limits as low as 2 cells/1 mL whole blood.^117^ Electrochemical detection methods have already demonstrated detection limits ranging from 2–7 cells per mL of whole blood, or 1 cell/1 mL in PBS supplemented with human mononuclear cells.^114^ Detection limits for optical means typically range from 1–50 cells per mL of whole blood,^41,56,115,116,118,120,121,139^ with physical methods somewhat poorer.^138,158^

### 4.1 Future directions in integrated CTC isolation & detection

While scalable and potentially very cost-effective integrated platforms hold great promise for supporting high capture efficiency and sensitivities high enough to detect as low as 1 cell per mL, such methods are early in development and have yet to translate into clinical practice. Reducing the turnaround time to less than 1 hour per sample (rather than the ∼1/2 day typically associated with a CellSearch analysis) and substantially lowering cost and increasing reliability would enable the application of this currently prohibitively expensive technique to routine clinical practice. While the long-standing optical method of detection *via* IF imaging is able to achieve low detection limits,^116,121^ this comes with inherent time challenges, as discussed in section 3.^116,121^

Methods of CTC isolation involving sample incubation with Ab-functionalised MNPs show promise due to their high capture efficiencies. However, reports to date have required off-chip sample incubation with MNPs, increasing processing time and the potential for cell loss with manual transfer.^115,117,118,120–122^ Thus, there is still need for development of more intimately integrated derivatives to increase throughput. Purely physical methods of CTC analysis/assaying remain rare, and are associated with relatively poor detection limits.^138,158^ While the long-standing optical method of detection *via* IF imaging sensitive,^116,121^ this comes with inherent time and throughput challenges, as discussed in section 3.^116,121^ Simple forms of optical assay such as brightfield^142^ or up-conversion luminescence imaging^118^ can be chip-integrated offer reduced labour and time, but these do not offer any phenotypic or profiling information about the captured CTCs. Reports discussed above which implemented innovative designs such as single-cell immunoblotting,^41^ rapid isothermal nucleic acid detection assay,^120^ and *in situ* Raman barcoding^113^ represent, then, the most interesting avenues for optical detection. Such methods offer phenotypic information through either multiplexing cell-surface marker analysis or uncovering intracellular protein expression. These approaches enable an on-chip classification of CTC subtype based on their expression of a range of established markers or receptors. This is particularly advantageous, as the clinician is given more than a mere binary *i.e.*, CTC positive/negative result, enabling more informed decisions on diagnosis or treatment avenues.

The integrated platforms in this review represent a significant advance down the road towards a routine low blood volume CTC analysis sufficiently robust to underpin new biological investigations and reliable assays. These analyses can reveal important insight into genomic markers of disease metastasis and ultimately inform clinical decision making.^115,122^ Future integrated devices could play a major role in conveniently sorting CTCs based on the cell surface expression of specific markers.^115,122^ Such platforms could facilitate downstream analysis and phenotypic evaluation by genomic profiling of, potentially, individual cells. Ultimately, we have sought to summarise the broad range of scalable and fully integrated toolboxes that are being developed. These may ultimately create opportunities for routine and convenient liquid biopsies and be applied in disease identification and staging, assessing progression risk, and guiding treatment.

## Author contributions

Sophia M. Abusamra: writing – original draft preparation, writing – review & editing. Robert Barber: writing – original draft preparation, writing –review & editing. Mohamed Sharafeldin: writing – original draft preparation, writing – review & editing. Claire M. Edwards: writing – review & editing, supervision. Jason J. Davis: writing – original draft preparation, writing – review & editing, supervision.

## Conflicts of interest

There are no conflicts of interest to declare.

## Supplementary Material
